# Nondestructive Detection of Sunflower Seed Vigor and Moisture Content Based on Hyperspectral Imaging and Chemometrics

**DOI:** 10.3390/foods13091320

**Published:** 2024-04-25

**Authors:** Peng Huang, Jinfu Yuan, Pan Yang, Futong Xiao, Yongpeng Zhao

**Affiliations:** College of Mechanical and Electrical Engineering, Sichuan Agriculture University, Ya’an 625014, China; 14130@sicau.edu.cn (P.H.); 2021317015@stu.sicau.edu.cn (J.Y.); 2022217010@stu.sicau.edu.cn (P.Y.); 2022317028@stu.sicau.edu.cn (F.X.)

**Keywords:** hyperspectral imaging technology, sunflower seed vitality, seed moisture content, feature extraction, ensemble learning model

## Abstract

Sunflower is an important crop, and the vitality and moisture content of sunflower seeds have an important influence on the sunflower’s planting and yield. By employing hyperspectral technology, the spectral characteristics of sunflower seeds within the wavelength range of 384–1034 nm were carefully analyzed with the aim of achieving effective prediction of seed vitality and moisture content. Firstly, the original hyperspectral data were subjected to preprocessing techniques such as Savitzky–Golay smoothing, standard normal variable correction (SNV), and multiplicative scatter correction (MSC) to effectively reduce noise interference, ensuring the accuracy and reliability of the data. Subsequently, principal component analysis (PCA), extreme gradient boosting (XGBoost), and stacked autoencoders (SAE) were utilized to extract key feature bands, enhancing the interpretability and predictive performance of the data. During the modeling phase, random forests (RFs) and LightGBM algorithms were separately employed to construct classification models for seed vitality and prediction models for moisture content. The experimental results demonstrated that the SG-SAE-LightGBM model exhibited outstanding performance in the classification task of sunflower seed vitality, achieving an accuracy rate of 98.65%. Meanwhile, the SNV-XGBoost-LightGBM model showed remarkable achievement in moisture content prediction, with a coefficient of determination ($R^{2}$) of 0.9715 and root mean square error (RMSE) of 0.8349. In conclusion, this study confirms that the fusion of hyperspectral technology and multivariate data analysis algorithms enables the accurate and rapid assessment of sunflower seed vitality and moisture content, providing robust tools and theoretical support for seed quality evaluation and agricultural production practices. Furthermore, this research not only expands the application of hyperspectral technology in unraveling the intrinsic vitality characteristics of sunflower seeds but also possesses significant theoretical and practical value.

## 1. Introduction

Seed vigor [1] plays a pivotal role in agricultural production and can be defined as the capability of seeds to maintain viability and germination potential [2]. Sunflower, being a significant economic crop due to its abundant oil resources and nutritional value in the food industry, livestock production, and beekeeping, holds an indispensable position in various domains [3]. The quality of seeds directly influences the production efficiency and output quality of sunflowers, with seed vigor and moisture content serving as crucial indicators of seed quality. Seed vigor, a sensitive parameter that reflects the physiological status and germination potential of seeds, not only reveals the strength of seed germination capacity but also uncovers the early-field-growth health and anticipated performance [4,5]. On the other hand, seed moisture content directly affects seed storage ability and germination efficiency, and achieving an appropriate moisture level becomes paramount to ensuring seed vitality and optimal germination performance (as mentioned above). During the storage stage, excessive seed moisture content stimulates enzymatic activity, intensifies respiration, and subsequently depletes the seed’s stored resources, leading to issues such as decay and mold [6]. Consequently, accurate determination of sunflower seed vigor and moisture content holds decisive significance in optimizing seed quality management and enhancing sunflower yield and quality.

Various methods are employed for the assessment of traditional seed viability and moisture content, encompassing techniques such as germination tests, permeability tests, color detection, conductivity determination, respiration metabolism analysis, and enzymatic activity analysis [7,8]. In these methods, viability assessment typically relies on the performance of seed germination under favorable conditions or indirect evaluation through physiological parameters such as conductivity, respiration rate, and specific enzymatic activity. These parameters profoundly reveal the physiological condition and vitality of the seeds. Regarding moisture content determination, commonly utilized approaches include distillation methods, drying methods [9], Soxhlet extraction [10], and Karl Fischer titration methods [11]. Although these conventional methods offer visual representations of seed viability and moisture content, they still present several limitations, such as prolonged testing cycles, low efficiency, high costs, cumbersome procedures, and even the potential for physical damage to the seeds. Given these limitations, the development of a rapid, nondestructive technique for assessing the viability and moisture content of sunflower seeds holds significant value in improving the efficiency of seed quality testing and protecting seed resources.

During the process of seed aging, changes occur in the external characteristics (such as color, transparency, surface structure, and optical response) as well as spectroscopic properties [12,13] due to the evolution of the internal tissue structure and chemical composition. Recent studies have extensively employed optical detection techniques, such as near-infrared spectroscopy (NIR), laser-induced fluorescence (LIF), and Raman spectroscopy, to explore various aspects including seed variety identification [14,15], phenolic compound content [16], mold condition [17], and vitality assessment [18,19], among others. However, NIR technology is constrained by single-point sampling and susceptible to the surface nonuniformity of samples. Although fluorescence spectroscopy can be used for specific marker detection, it requires additional processing steps that may introduce harmful substances. Raman spectroscopy is highly sensitive to humidity, especially in the presence of water molecules, and cannot accurately determine moisture content.

Hyperspectral technology has gained extensive utilization in the field of nondestructive and rapid assessment of seed vitality owing to its inherent characteristics of rich multispectral information, high spatial resolution, and strong detection sensitivity [20,21,22,23,24]. Furthermore, groundbreaking research by Wang, Zheli et al. [25] ingeniously integrated hyperspectral imaging technology with machine learning algorithms, facilitating precise identification and classification of deteriorated maize seeds through meticulous analysis of surface texture features. The pioneering study provides a robust methodological foundation for the innovative enhancement of seed inspection devices. However, due to the exorbitant costs associated with collection and maintenance, the complex requirements for data processing, and the impact of environmental factors on imaging accuracy, it confronts inherent limitations. To surmount these constraints, it becomes imperative to address these challenges through technological optimization, research and development of cost-effective equipment, and the implementation of efficient algorithms.

Conventional hyperspectral techniques primarily focus on the spectral response of seeds, often overlooking the fundamental intrinsic physicochemical characteristics that determine their optical properties. Recent research trends aim to integrate hyperspectral imaging with chemometrics methods to unravel the connection between the internal physicochemical attributes of seeds and their optical manifestations, thereby achieving a more comprehensive understanding of seed transformation processes [26,27]. Through a comprehensive analysis of both the internal and external features of seeds, a more accurate assessment of their quality and characteristics can be achieved, providing a more scientifically informed basis for seed production and management decisions. Drawing inspiration from such methodologies, Wang, Zheli et al. [28] successfully employed long-wavelength near-infrared hyperspectral imaging technology (LWNIR HIS) to accurately predict the moisture content of individual maize seeds, enabling real-time monitoring of seed quality. This achievement holds significant implications for seed quality assessment and agricultural production practices. It is noteworthy that the spectral range of short-wavelength near-infrared hyperspectral imaging (SWNIR HIS) typically spans from 400 to 1600 nm, encompassing the primary absorption bands of various vital biomolecules within the seeds, including moisture, proteins, lipids, and carbohydrates. These components are intricately linked to seed vigor and moisture content. In comparison to LWNIR HIS, SWNIR light exhibits greater penetration [29], thereby improving the signal-to-noise ratio of spectral data and facilitating the deciphering of spectral characteristics. This, in turn, enables the establishment of robust correlation models between these characteristics and intrinsic quality parameters. Accordingly, our study seeks to utilize hyperspectral imaging technology (HSI) to monitor the optical characteristics changes in sunflower seeds during artificial aging. This aims to establish a model for accurately determining the moisture content of sunflower seeds, thus deepening our understanding and evaluation of sunflower seed quality.

This study aims to utilize hyperspectral imaging technology to explore the inherent quality of sunflower seeds at different stages of aging and to establish a quantitative analysis model for the microscale water content of seeds. The core objectives of this research are as follows: (1) to develop a vitality classification model for sunflower seeds based on standard germination test data guided by hyperspectral imaging; (2) to establish a corresponding predictive regression model for sunflower seed water content using the classical drying method guided by hyperspectral imaging; (3) to delve into the correlation between hyperspectral features, seed vitality indices, and water content. By achieving the aforementioned goals, this study will further uncover the profound potential of hyperspectral imaging technology in seed quality assessment and provide a more comprehensive and precise tool for evaluating seed quality in agricultural production. The outcomes of this research not only enrich the methodological framework for seed quality analysis but also hold significant theoretical and practical implications for the seed industry and modern agricultural production.

## 2. Materials and Methods

### 2.1. Seed Sample Preparation

In order to acquire sunflower seed samples exhibiting varying levels of vitality, this research employed a methodology of artificially induced aging, given the consistency between artificial and natural aging at the seed metabolic level [30,31]. In the year 2022, a purchase was made of 200 g of sunflower seeds of the “Mao Hua Oil Giant” variety, produced by “Zhongke Maohua”. From this batch, a total of 500 seeds without any signs of mold or damage were carefully selected and divided into five batches, each containing 100 seeds. One of the groups served as the control group, labeled as fresh seeds with no storage (NAA), and was kept in standard indoor conditions (temperature 25 °C, relative humidity 30%). The remaining four groups of seeds were sequentially placed in a seed-aging chamber, where they underwent graded aging treatments lasting for 2 days (1AA), 4 days (2AA), 6 days (3AA), and 8 days (4AA) under constant conditions (temperature 45 °C, humidity 90%). This process allowed the creation of a curated collection of sunflower seed samples covering five distinct vitality gradients. Following the completion of the aging treatments, the seeds were left to rest at room temperature for two days, ensuring a uniform internal moisture content across all groups. Subsequently, the seeds were brought to the laboratory for the acquisition of hyperspectral image data.

### 2.2. Hyperspectral Image Data Acquisition

This study employed the GaiaSorter hyperspectral sorting system, manufactured by Zolix Instruments Co., Ltd., Beijing, China. The system was equipped with an Image-λ “spectral imaging” series high-spectral camera, specifically the Image-λ-V10E-LU model. The spectral range for data acquisition covered 384–1034 nm, with a spectral sampling interval of 2.8 nm, utilizing the built-in push-scan scanning mode. The lens of this spectral imaging device captured the high-spectral data of seed samples along with corresponding images for each wavelength band. The system was programmed to collect 50 seed samples in each instance, resulting in a total of 500 samples collected throughout this study.

In order to minimize the impact of fluctuations in light source temperature on the experimental results, the instrument was preheated for a duration of 30 min prior to the commencement of the experiment, allowing it to attain a state of stability. Furthermore, considering the differential light absorption characteristics associated with various colors and the influence of background effects, an initial acquisition consisted of capturing a reference dark-field image and an all-white calibration image, which served to correct the spectral data. Following the completion of sample collection, the acquired data were imported into the SpecVIEW software for grayscale calibration, employing the standardized procedure outlined by Equation (1) in accordance with best practices.

(1) $$X_{\mathrm{ref}\;}=\frac{X_{\mathrm{raw}\;}-X_{\mathrm{dark}\;}}{X_{\mathrm{white}\;}-X_{\mathrm{dark}\;}}$$

In the formula, $X_{\mathrm{ref}}$ represents the high-spectral data after monochrome correction, $X_{\mathrm{raw}\;}$ denotes the original high-spectral image, $X_{\mathrm{dark}\;}$ stands for the dark background data obtained by covering the camera lens, and $X_{\mathrm{white}\;}$ is the full white calibration image obtained by placing a standard white board with 100% reflectance at the same distance as the measured object.

**Figure 1:**
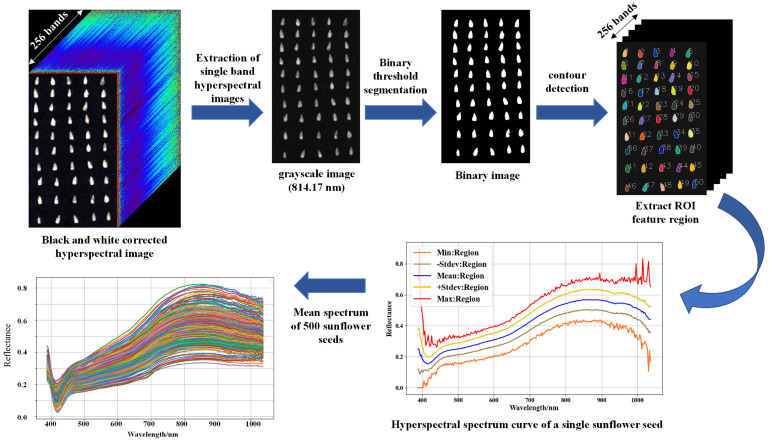
Flow chart of extracting spectral curves from hyperspectral images of sunflower seeds.

The calibrated hyperspectral image data were subjected to spectral analysis using the professional software ENVI 5.3, as depicted in Figure 1. To mitigate the influence of the background on the spectral reflectance of sunflower seeds, the region of interest (ROI) technique was employed, whereby the overall area of each individual sunflower seed sample was precisely extracted to calculate the average spectral reflectance. By analyzing the full spectral range image, a single-band grayscale image (814.17 nm) was carefully selected for its distinctive spectral reflectance contrast between the seeds and the background. Subsequently, the image was converted into a binary format through thresholding, yielding a black-and-white representation. Employing the threshold segmentation method, the seed contours were accurately extracted from the binary image, resulting in the successful acquisition of 500 distinct regions of interest (ROIs) encompassing individual sunflower seeds.

### 2.3. Standard Germination Test

To evaluate the vitality of sunflower seeds based on high-spectral imaging data, this study strictly followed the standardized germination test procedure established by the International Seed Testing Association (ISTA) [32]. At the outset of the experiment, thorough cleaning and disinfection of the seeds and germination plates were performed using a 3% hypochlorous acid solution, followed by natural air drying at room temperature. The experiment utilized filter paper with a pH range of 6.0 to 7.5 as the germination medium, employing the method of double-layered paper germination (BP), wherein the seeds were placed in the center between two layers of filter paper. Following the sequence of numbering during high-spectral data collection, the sunflower seeds were individually arranged in their corresponding germination plates, which were then placed in an intelligent constant temperature and humidity chamber for germination under constant conditions of 25 °C temperature and 40% humidity. Throughout the germination period, water was replaced every 6 h to ensure that the filter paper remained suitably moist. On the fourth day of germination, the initial sprouting quantity was recorded, and on the tenth day, the final counting was performed, thereby determining key indicators such as germination rate and seedling length for each vitality gradient of sunflower seeds. Data processing was conducted using the following calculation formula:

(2) $$\;\mathrm{Germination}\;\mathrm{potential}\;\left(GP\right)=\frac{n_{1}}{N}\times 100\%$$

(3) $$\;\mathrm{Germination}\;\mathrm{rate}\left(GR\right)=\frac{n_{2}}{N}\times 100\%$$

(4) $$\;\mathrm{Germination}\;\mathrm{index}\;\left(GI\right)=\sum \frac{G_{t}}{D_{t}}$$

(5) Vigorindex(VI)=GI×S

In the aforementioned equations, $n_{1}$ denotes the number of germinated sunflower seeds on the 4th day, $n_{2}$ represents the number of germinated sunflower seeds on the 10th day, $G_{t}$ signifies the daily germination count within the first 10 days of the germination test, $D_{t}$ indicates the number of germination days, and *S* denotes the average shoot length (cm) of the seedlings on the 10th day.

### 2.4. Moisture Content Determination

Sunflower seeds, from which hyperspectral image data were collected, were weighed using a high-precision electronic balance (FA1004, Qun ’an Scientific Instruments (Zhejiang) Co., Ltd., Huzhou, China) with an accuracy of 0.1 mg. The weighed seeds were then labeled and placed into an aluminum container, which was subsequently transferred to a drying oven set at a temperature of 103 °C ± 1 °C for a period of 48 h until a constant mass was achieved. The moisture content of the seeds was subsequently calculated based on Equation (6).

(6) $$\;\mathrm{Moisture}\;\mathrm{content}\;\left(MC\right)=\frac{m_{0}-m_{1}}{m_{0}-m}\times 100\%$$

where $m_{0}$ represents the mass of an individual seed with the aluminum shell before drying, $m_{1}$ represents the mass of an individual seed with the aluminum shell after drying, and *m* represents the mass of the aluminum box.

### 2.5. Data Analysis Method

#### 2.5.1. Spectral Preprocessing

The raw hyperspectral data are susceptible to various disturbances, such as environmental interference and instrument instability, which introduce noise and outliers. To enhance the quality and reliability of the data, this study employed a suite of preprocessing techniques for data purification. Specifically, we employed the Savitzky–Golay smoothing technique (SG) [33], renowned for its inherent ability to effectively smooth spectral curves while efficiently mitigating high-frequency noise interference. Compared to conventional moving-average smoothing methods, the SG technique has exhibited remarkable advantages in preserving crucial data features, such as peaks and valleys, thereby being particularly well suited for meticulous analysis of agricultural characteristics, including seed viability and moisture content. Simultaneously, we utilized the standard normal variate correction method (SNV) [34] to rectify systematic errors and normalize the distribution of the data. Furthermore, the multiscatter correction (MSC) technique [35] was employed to effectively eliminate scattering effects in the spectral data, thereby ensuring the precision and credibility of the original hyperspectral image data.

#### 2.5.2. Sample Partition

Segmenting hyperspectral data into training and testing sets allows for model training and performance evaluation. In this study, the SPXY (sample set partitioning based on joint x–y distances) algorithm [36] was employed. This approach is an exemplary method for sample set partitioning, as it utilizes the joint x–y distance measure based on the Euclidean distance (Equation (7)) to quantify the distances between samples.

(7) $$d_{x}\left(p,q\right)=\sqrt{\sum_{i=1}^{n}{\left(x_{p}\left(i\right)-x_{q}\left(i\right)\right)}^{2}}$$

In the equation, $x_{p}$ and $x_{q}$ represent two distinct sample datasets, while *n* denotes the quantity of spectral bands.

Subsequently, the algorithm partitions the sample set into different subsets based on the distances between samples. The principle of partitioning is to assign adjacent samples to the same subset, ensuring that the samples within each subset are closer to each other in the x–y plane. The calculation formula is as follows:

(8) $$d_{y}\left(p,q\right)=\left|y_{p}-y_{q}\right|$$

(9) $$d_{xy}\left(p,q\right)=\frac{d_{x}\left(p,q\right)}{\max_{p,q}d_{x}\left(p,q\right)}+\frac{d_{y}\left(p,q\right)}{\max_{p,q}d_{y}\left(p,q\right)}$$

In the formula, $d_{x}\left(p,q\right)$ represents the Euclidean distance between spectra, while $d_{y}\left(p,q\right)$ represents the Euclidean distance between physical and chemical measurement values. The objective of this approach is to preserve the structural features of the original data as much as possible, thereby ensuring that the distribution of the partitioned subsets on the x–y plane becomes more uniform and compact.

#### 2.5.3. Feature Dimension Reduction Algorithm

Due to the multitude of spectral bands (256 in total) present in the acquired hyperspectral data, there exists a considerable level of intrinsic redundancy. This consequently leads to lengthy processing times and high computational complexity. Therefore, prior to constructing classification and regression models, it is imperative to effectively extract and optimize the hyperspectral features, with the aim of eliminating redundant information and reducing data dimensions. By doing so, we can circumvent the challenges associated with the subsequent model construction and analysis, including prolonged training times, prediction delays, and limited generalization performance arising from the high-dimensional nature of the data. In this paper, we employ principal component analysis (PCA), extreme gradient boosting (XGBoost), and stacked autoencoder (SAE) algorithms to refine the wavelength dimensions of the features, thereby mitigating the computational complexity of the models.

Principal component analysis (PCA), an extensively employed unsupervised linear dimensionality reduction technique [37], operates on the fundamental principle of transforming correlated variables within the original dataset into a set of mutually orthogonal principal components. These principal components are ranked in descending order based on their respective abilities to explain variance; while preserving the essential characteristics of the data, PCA strives to maximize the projected variance in the reduced subspace. This effectively compresses redundant data and unveils pivotal information.

Extreme gradient boosting (XGBoost), a powerful gradient boosting algorithm, showcases efficient, precise, and highly scalable feature dimension reduction performance through the integration of gradient optimization principles, regularization techniques, and parallel tree model construction mechanisms [38,39]. With its outstanding practicality, this algorithm has played a crucial role in feature selection, dimension reduction, classification, regression analysis, and feature ranking, making it widely employed in the feature dimension reduction phase of multidimensional data mining tasks.

The autoencoder (AE), serving as a neural network model, primarily focuses on the encoding and decoding processes of data, emulating the intricacies of data compression and reconstruction. This model encompasses an encoder component that maps input data into a lower-dimensional latent space representation, thereafter utilizing a decoder component to reconstruct the original input from this lower-dimensional representation [40]. The fundamental concept of the AE lies in the pursuit of learning the most discriminative compressed representation of the input data, thereby offering an effective tool for tasks such as dimensionality reduction, denoising, and feature learning.

**Figure 2:**
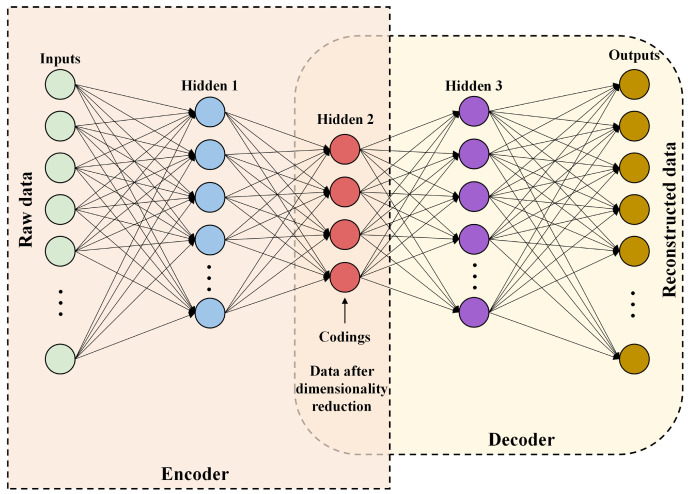
Schematic diagram of stacked autoencoder.

In the process of dimensionality reduction in an SAE, as shown in Figure 2, there are typically two crucial steps: Firstly, the pretraining phase is where each layer is individually trained as a single-layer autoencoder, serving to initialize weights and biases and to learn preliminary meaningful feature representations. Secondly, the fine-tuning phase involves consolidating the pretrained layers into a stacked autoencoder, optimizing the entire network using backpropagation and gradient descent algorithms, aiming to minimize reconstruction errors, thus ensuring the effective acquisition of hierarchical feature expressions from the data.

#### 2.5.4. Modeling Method

Random forest (RF) is an ensemble learning technique that leverages the construction and integration of multiple decision trees to facilitate predictions. By employing a voting or averaging strategy to amalgamate the outputs of individual decision trees, RF enhances the precision of model predictions. During the construction of each decision tree, the random forest method incorporates a random sampling mechanism involving nonreplacement sampling of training samples and features. This process effectively mitigates the risk of overfitting and bolsters the generalization capabilities of the model [41].

Light gradient boosting machine (LightGBM), a decision tree ensemble model built on a gradient boosting framework, employs the histogram-based tree learning algorithm to significantly enhance training efficiency [42]. This algorithm introduces the leaf-wise growth strategy, also known as the best–first strategy, with the aim of optimizing the balance between model complexity and predictive accuracy [43]. Furthermore, LightGBM integrates gradient-based one-sided sampling and feature parallel learning techniques, aiming to further accelerate model training speed and enhance model predictive precision.

#### 2.5.5. Model Evaluation Metrics

This study employs a series of widely recognized performance evaluation metrics to assess the predictive capabilities of the model. These metrics include accuracy, training set coefficient of determination ($R_{c}^{2}$), root mean square error of the training set (RMSEC), testing set coefficient of determination $(R_{p}^{2}$), and root mean square error of the testing set (RMSEP) [44,45]. Accuracy measures the proportion of correctly classified samples out of the total number of samples, reflecting the model’s precision in classification tasks. The coefficient of determination ($R^{2}$) is used to indicate the correlation between the model’s predicted values and the actual observed values, ranging from 0 to 1. A higher value signifies superior predictive performance of the model. Root mean square error (RMSE), on the other hand, is employed to evaluate the disparity between the model’s predicted values and the actual values. A smaller RMSE indicates a smaller prediction deviation and thus a more optimal performance of the model.

(10) $$\mathrm{Accuracy}=\frac{TP+TN}{TP+TN+FP+FN}\times 100\%$$

(11) $$R_{c}^{2}=1-\frac{\sum_{i=1}^{n_{c}}{\left(y_{i,c}-{\hat{y}}_{i,c}\right)}^{2}}{\sum_{i=1}^{n_{c}}{\left(y_{i,c}-{\bar{y}}_{c}\right)}^{2}}$$

(12) $$R_{p}^{2}=1-\frac{\sum_{i=1}^{n_{p}}{\left(y_{i,p}-{\hat{y}}_{i,p}\right)}^{2}}{\sum_{i=1}^{n_{p}}{\left(y_{i,p}-{\bar{y}}_{p}\right)}^{2}}$$

(13) $$RMSEC=\sqrt{\frac{1}{n_{c}}\sum_{i=1}^{n_{c}}{\left(y_{i,c}-{\hat{y}}_{i,c}\right)}^{2}}$$

(14) $$RMSEP=\sqrt{\frac{1}{n_{p}}\sum_{i=1}^{n_{p}}{\left(y_{i,p}-{\hat{y}}_{i,p}\right)}^{2}}$$

In the equations, true positive (TP) represents the quantity of positively classified samples that the model accurately predicts as positive, true negative (TN) represents the quantity of negatively classified samples that the model accurately predicts as negative, false positive (FP) represents the quantity of negatively classified samples that the model inaccurately predicts as positive, and false negative (FN) represents the quantity of positively classified samples that the model inaccurately predicts as negative. $n_{c}$ is the number of samples in the training set, $n_{p}$ is the number of samples in the prediction set, $y_{i,c}$ is the measured value of the *i* sample in the training set, ${\hat{y}}_{i,c}$ is the predicted value of the *i* sample in the training set, $y_{i,p}$ is the measured value of the *i* sample in the test set, ${\hat{y}}_{i,p}$ is the predicted value of the *i* sample in the test set, ${\bar{y}}_{c}$ is the mean value of the training set samples, and ${\bar{y}}_{p}$ is the mean value of the test set samples.

## 3. Results and Discussion

### 3.1. Standard Germination Test Results

Table 1 presents the vitality indicators of sunflower seeds at different stages of aging. It is evident that as the aging time increases, the quantity of sprouted samples significantly exhibits a diminishing trend. Correspondingly, the germination potential, germination rate, germination index, and vitality index also display a declining pattern. These findings elucidate that the artificial aging treatment alters certain physicochemical parameters within the seeds, leading to the loss of vitality in a subset of sunflower seeds.

In this study, it was observed that due to the artificial experimental conditions, one seed in the NAA group and three seeds in the 1AA group suffered damage. The NAA group consisted of helianthus seeds that had not undergone an aging process, while the 1AA group comprised helianthus seeds aged for 2 days. Additionally, aging durations of 4, 6, and 8 days were associated with the 2AA, 3AA, and 4AA groups, respectively.

### 3.2. Results of Moisture Content Determination

**Figure 3:**
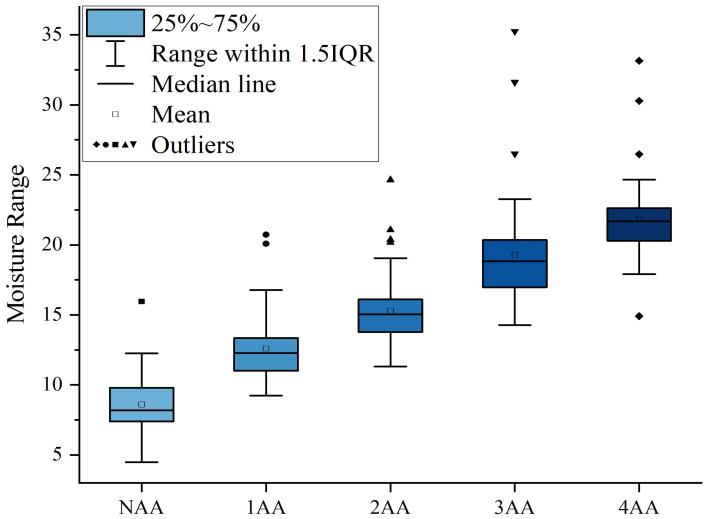
Box plot for outlier detection of moisture content in sunflower seeds.

We used box plots for outlier detection of sunflower seeds in five groups, with the detection results shown in Figure 3.

The box plot, a visual representation of the distribution characteristics of moisture content, showcases the median, quartiles, and extreme values of five different groups. It unveils the nuanced variations in moisture content across these groups. Any data points surpassing 1.5 times the interquartile range are considered outliers and subsequently excluded from further analysis. After excluding 14 such outliers, the revised statistical data on sunflower seed moisture content are succinctly summarized in Table 2.

### 3.3. Original Spectrum and Pretreatment Results

**Figure 4:**
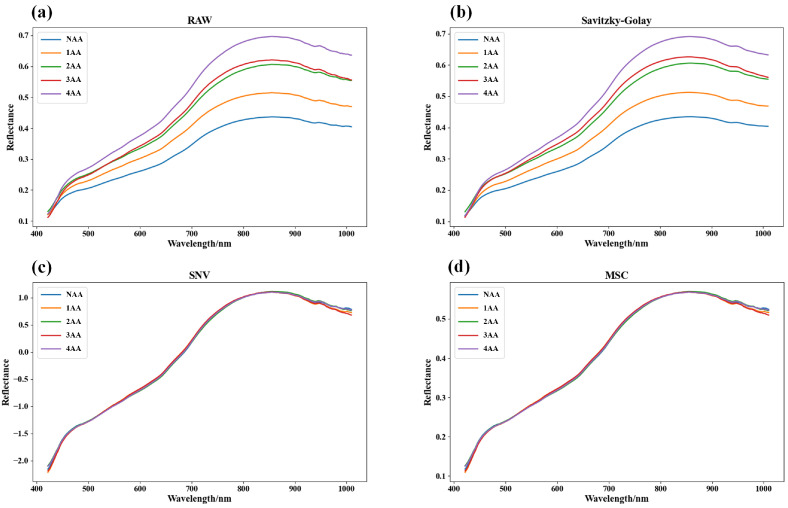
Average spectra of sunflower seed samples of different aging categories: (**a**) original average spectra; (**b**) SG smoothing pretreatment average spectrum; (**c**) SNV pretreatment average spectrum; (**d**) MSC pretreated mean spectrum.

Due to the influence of instrumental noise, systematic biases, and other adverse factors during the process of hyperspectral measurement, the original spectra commonly exhibit significant noise in the short- and long-wave regions. Hence, for analysis purposes, this study selectively utilized 232 effective spectral bands ranging from 421.87 nm to 1009.44 nm. Figure 4 depicts the original average spectra and the preprocessed spectra of sunflower seed samples from different aging levels. It can be observed that after the application of SNV and MSC preprocessing techniques, the spectral differences among seed categories were somewhat reduced, suggesting that these two preprocessing methods may not effectively emphasize classification boundaries in the task of seed vitality classification. Conversely, the SG preprocessing method successfully maintained the spectral distinctiveness of different aging levels, potentially enhancing performance in seed vitality classification.

In the natural senescence process of sunflower seeds, the internal nutritional components undergo lipid oxidation, leading to an intensified rate of degradation of nutrient substances as aging time prolongs. As depicted in Figure 4, an exploration of the average spectral curves of sunflower seeds at different stages of aging reveals a consistent increasing trend with the progression of senescence. Substantial differences in spectral curves between newly formed seeds and those with varying degrees of aging are likely attributable to the ongoing decomposition of fatty acids, proteins, and carbohydrates within the seeds, resulting in the generation of free radicals and oxidation products. These changes manifest as significant enhancements in spectral reflectance [46].

### 3.4. Feature Dimension Reduction Results

#### 3.4.1. PCA

**Figure 5:**
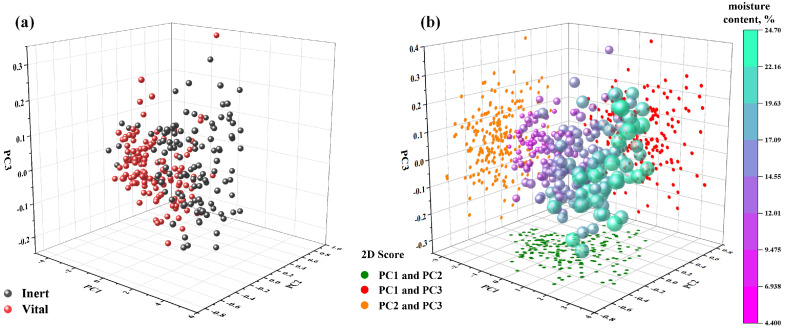
(**a**) PCA 3D distribution of sunflower seed vigor; (**b**) PCA 3D distribution of moisture content of sunflower seeds.

Utilizing principal component analysis (PCA), an in-depth analysis was conducted on the average spectral reflectance data of sunflower seeds’ raw spectral data within the wavelength range of 420 to 1010 nm. This analysis aimed to extract the top three principal components that contribute significantly to the overall variability. As depicted in Figure 5, the three-dimensional PCA score plot visually portrays the comprehensive representation of these principal components in capturing the essence of the original spectral information.

Upon analysis of Figure 5, it becomes evident that the first three principal components succinctly encapsulate the vast majority of the spectral information pertaining to sunflower seeds. In particular, Figure 5a depicts the concentration of vitality data, wherein the cumulative contribution of the top three principal components amounts to an impressive 99.90%, with PC1 accounting for 97.65%, PC2 for 1.96%, and PC3 for 0.29%. Despite the subtle overlap between vital and nonvital seeds within the principal component space, the existing classification tasks are confronted with a certain level of challenge.

On the other hand, Figure 5b showcases the moisture content dataset, whose top three principal components cumulatively contribute to 99.86%, specifically corresponding to 94.69% for PC1, 4.35% for PC2, and 0.82% for PC3. Similarly, employing analogous methods, one can conduct principal component analysis on the preprocessed spectral curves, thus extracting the corresponding weight coefficients.

#### 3.4.2. XGBoost

**Figure 6:**
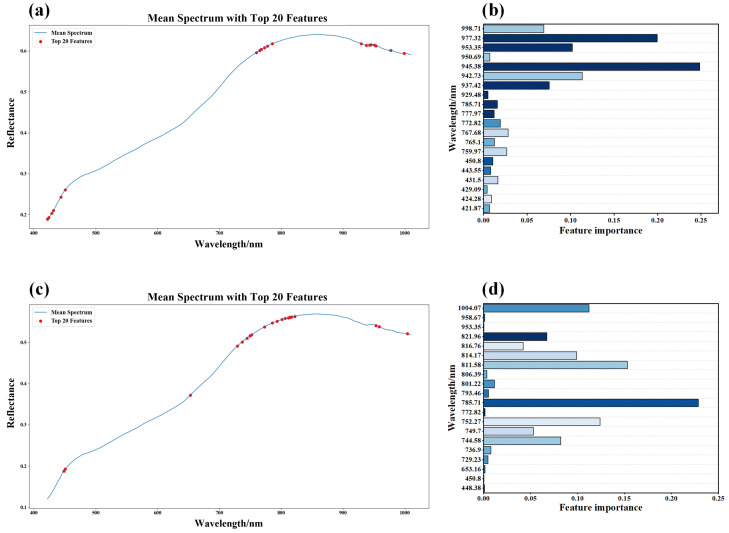
Top 20 feature wavelengths extracted by XGBoost algorithm: (**a**) feature bands extracted by classification model; (**b**) XGBoost feature band extraction weight map; (**c**) feature band extraction diagram of regression model; (**d**) XGBoost feature band extraction weight map.

After performing feature extraction using the XGBoost algorithm on the dataset of sunflower seed vitality classification and water content regression, the distribution of importance for the top 20 feature bands is extensively depicted in Figure 6. It is noteworthy that when only the top 20 feature bands with the highest contribution are selected, the cumulative contribution rate of these bands has already reached 99.97%. This implies that the extracted key features encompass nearly all vital information, exemplifying their eminence.

An analysis of Figure 6a,c reveals that the XGBoost algorithm extracts significant spectral bands primarily concentrated within the ranges of 420–600 nm and 700–1010 nm for vitality classification and moisture content prediction datasets of sunflower seeds. Of particular interest, as depicted in Figure 6b, the top five contributing feature bands for vitality classification are 945.38 nm, 977.32 nm, 942.73 nm, 953.35 nm, and 937.42 nm, primarily located within the 900–1010 nm range. It is worth noting that the spectral reflectance within this range exhibits a close correlation with biologically active substances such as amino acids, lactose, and enzymes [47].

Similarly, insights from Figure 6d demonstrate that the five key feature bands for moisture content prediction are 785.71 nm, 811.58 nm, 752.27 nm, 1004.07 nm, and 814.17 nm, mainly distributed within the 700–900 nm range. This spectral band exhibits significant correlations with pigments, water content, and protein levels [48,49].

#### 3.4.3. Stacked Autoencoder

**Figure 7:**
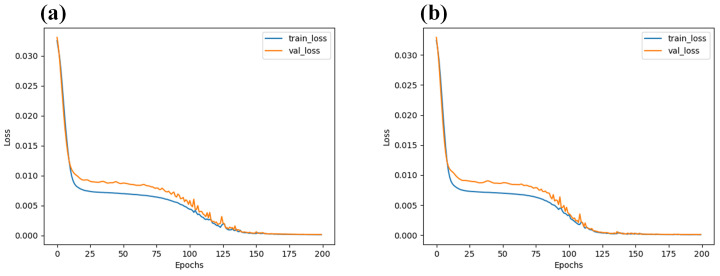
Loss diagram after extraction of 20 dimensions by SAE algorithm: (**a**) original spectra of sunflower seed vitality; (**b**) original spectra of moisture content of sunflower seeds.

By employing the stack autoencoder (SAE) algorithm, the average spectra of the classification of sunflower seed vitality and regression of moisture content were subjected to a feature dimension reduction, as depicted in Figure 7. This study revealed that by reducing the dimensions to 20, after 150 training iterations, the reconstructed spectral features from the encoding process demonstrated an almost flawless restoration to their original counterparts. The loss, which measures the discrepancy between the encoded and original spectral features, was observed to be lower than 0.005. Such outcomes undeniably underscore the remarkable efficacy and potential of the stack autoencoder in the realm of spectral data dimensionality reduction.

Through a progressive greedy training process, the unsupervised learning framework of stacked autoencoders gradually uncovers and captures the deep-seated and intricate structural features within the dataset, facilitating the approximate reconstruction of the original data. In the context of spectral data analysis, this dimensionality reduction technique exemplifies both efficiency and rationality. By reducing the data dimensionality, it successfully distills crucial feature information while preserving essential data essence. Consequently, it offers a compact yet information-rich data representation, serving as a valuable prerequisite for subsequent analysis and processing procedures.

### 3.5. Classification Results of Seed Vigor

Table 3 presents the modeling accuracy results of various models after preprocessing and feature wavelength extraction. In this study, we employed a criterion for determining the vitality of sunflower seeds, which considers embryos with a length exceeding 2 centimeters within a 10-day period as vigorous and those falling short as nonvigorous. Utilizing the SPXY algorithm, a total of 246 sunflower seeds that successfully completed germination tests were allocated to training and prediction sets in a 7:3 ratio to assess the performance of the models in both training and prediction. The training set consisted of 172 seeds, among which 93 were classified as vigorous and 79 as nonvigorous. Conversely, the prediction set consisted of 74 seeds, with 36 classified as vigorous and 38 as nonvigorous.

By comparing and analyzing the original spectra with the modeling results obtained through different preprocessing algorithms, it becomes evident that the original spectra and SG smoothing preprocessing, due to their minimal transformation of the hyperspectral curves of seeds, do not alter the separability of spectral curves among different aging classes. Consequently, they demonstrate superior classification performance. On the other hand, the SNV and MSC preprocessing algorithms excessively correct the signals associated with seed biochemical properties. Although they eliminate overall spectral tilt and amplitude variations, they diminish the separability of characteristic peaks that initially exhibited significant intensity differences. Therefore, the performance of SNV and MSC preprocessing algorithms on the seed vitality classification dataset is less satisfactory.

Upon comparing the modeling results of the unprocessed full-spectrum data and the model utilizing feature dimensionality reduction, it becomes apparent that while the full-spectrum modeling captures effective information characteristics, the high-dimensional attributes of the original spectral data can lead to a propensity for model overfitting, thereby limiting its generalization performance on unknown test sets. In contrast, feature dimensionality reduction techniques significantly reduce the complexity of the dataset, thus facilitating the construction of more concise and easily generalizable classification models.

On the training set, both the XGBoost and SAE algorithms, after implementing feature dimensionality reduction, manage to preserve the effectiveness of the original spectral information to a certain extent, but they also exhibit a potential inclination toward overfitting the training data. Conversely, the PCA algorithm maximally reduces the data dimensionality, yet it may fail to fully elucidate the complete information content of the original spectra, resulting in a lack of significant advantage in classification performance.

Hence, the quest for feature selection or dimensionality reduction methods that effectively reduce dimensionality while preserving the crucial information becomes crucial. By doing so, we can prevent overfitting and simultaneously enhance the classification efficacy of the models on new samples.

In the experimental observation of the RF model, the inherent complexity of the model manifests in pronounced overfitting tendencies. This is evidenced by the remarkably high training accuracy of 99.42%, contrasted by a substantial decline to 90.54% in testing accuracy. Furthermore, the prolonged training time further corroborates the increased computational complexity of the model. In contrast, the LightGBM model effectively mitigates the overfitting issue through optimization algorithms, thereby demonstrating enhanced generalization performance.

**Figure 8:**
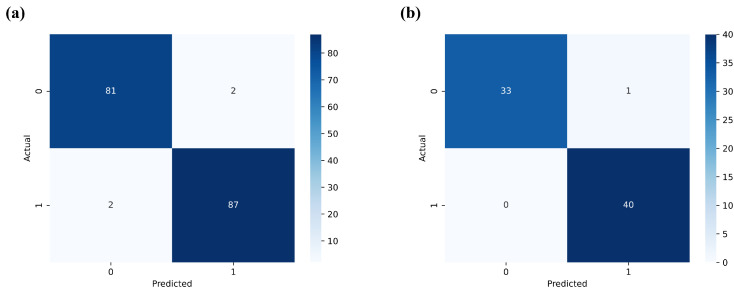
SG-SAE-LightGBM confusion matrix: (**a**) training set confusion matrix; (**b**) test set confusion matrix.

Empirical evidence confirms that by employing the SG preprocessing technique and SAE feature dimensionality reduction strategy, LightGBM achieves an accuracy of 97.67% on the training set, which further improves to 98.65% on the testing set. The confusion matrix of the SG-SAE-LightGBM algorithm, as depicted in Figure 8, reveals that it accurately classifies 73 instances of sunflower seed vitality, with only one misclassification. This phenomenon not only indicates LightGBM’s precise prediction capabilities in sunflower seed vitality classification tasks but also effectively reduces model complexity and overfitting risks. Consequently, it enhances predictive stability on unknown samples.

### 3.6. Prediction Results of Seed Moisture Content

This study employed a sample set of 236 sunflower seeds, using their moisture content data. The SPXY algorithm was utilized to allocate the training set (166 seeds) and the prediction set (70 seeds) in a 7:3 ratio. The full-spectrum data, along with the feature variables obtained through three feature extraction algorithms, were inputted separately into the RF (random forest) and LightGBM models. This allowed for the evaluation and comparison of the models’ performance in predicting seed moisture content. The modeling results are meticulously documented in Table 4.

The data presented in Table 4 exemplify the substantial enhancement of the performance of the regression model on the test set due to the implemented preprocessing steps. These findings shed light on the advantageous role of preprocessing methods in reducing spectral data noise and improving data purity. Notably, the SNV and MSC preprocessing algorithms demonstrate remarkable efficacy in this regard, underscoring their ability to effectively separate nonchemometric variations within the spectral data and thus accentuate the chemical information attributes closely linked to seed moisture content.

**Figure 9:**
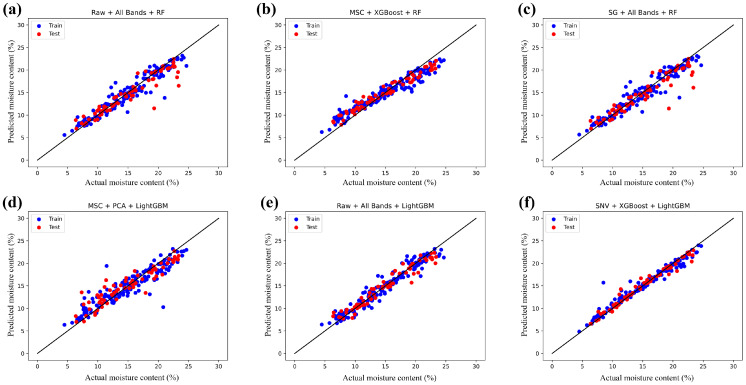
Sunflower seed moisture content prediction scatter plot: (**a**) Raw-All Bands-RF model; (**b**) MSC-XGBoost-RF model; (**c**) SG-All Bands-RF model; (**d**) MSC-PCA-LightGBM model; (**e**) Raw-All Bands-LightGBM model; (**f**) SNV-XGBoost-LightGBM model.

Comparatively, the RF model without preprocessing achieved a maximum coefficient of determination ($R_{p}^{2}$) of 0.8644 on the test set, as depicted in Figure 9a within the predictive scatter plot. However, applying the MSC preprocessing technique further elevated the model’s performance, as evidenced by an increased $R_{p}^{2}$ value of 0.9109. The corresponding predictive scatter plot is illustrated in Figure 9b. These outcomes robustly validate the indispensability of preprocessing in enhancing both the accuracy and the stability of seed moisture content prediction.

By comparing the analysis of full spectral data with the modeling results after dimensionality reduction, it was observed that although the full spectral modeling performed exceptionally well on the training set, as depicted in Figure 9c, with higher values of correlation coefficient ($R_{c}^{2}$) and root mean square error (RMSEC) compared to the validation set’s correlation coefficient ($R_{p}^{2}$) and test set root mean square error (RMSEP), this also revealed a significant risk of overfitting in the full spectral model. Conversely, the regression model using dimensionality reduction algorithms effectively addressed the issue of overfitting. However, the performance of the model after applying the PCA algorithm for dimensionality reduction was not satisfactory, as shown in Figure 9d, indicating that PCA to some extent failed to adequately preserve the original spectral information, leading to a significant loss of data information. In contrast, employing the XGBoost and SAE algorithms for dimensionality reduction of the spectral data not only reduced model complexity and improved training efficiency but also maximized the retention of the original spectral information, effectively mitigating the risks associated with overfitting.

Upon comparing the disparities in predictive performance between the RF and LightGBM models, it becomes evident that the RF model, owing to its heightened model complexity and potential for overfitting, coupled with its substantial resource requirements, struggles to achieve efficient and precise predictions. Conversely, the LightGBM model thrives on its lightweight characteristics, effortlessly achieving rapid regression predictions with fewer training parameters. Notably, on the unprocessed full-band original dataset, the LightGBM model has already showcased superior performance compared to the RF model, as depicted in Figure 9e. The correlation coefficients ($R_{c}^{2}$, $R_{p}^{2}$) for the training and validation sets are 0.9415 and 0.9416, respectively, while the root mean square errors (RMSEC, RMSEP) for the training set and testing set are 1.1894 and 1.1952, respectively. Furthermore, with the implementation of SNV preprocessing and XGBoost dimensionality reduction techniques, the predictive capabilities of the LightGBM model are significantly amplified. Notably, in Figure 9f, the correlation coefficients and root mean square errors achieve remarkable values of 0.9605, 0.9715 ($R_{c}^{2}$, $R_{p}^{2}$), and 0.9776, 0.8349 (RMSEC, RMSEP), respectively. Consequently, the LightGBM model demonstrates a striking advantage over the RF model in the prediction of sunflower seed moisture content, elevating not only the accuracy of predictions but also its practicality and reliability.

### 3.7. Correlation Analysis

In this study, the XGBoost algorithm was utilized to discern the top five key feature bands from the dataset analyzing sunflower seed vitality classification and moisture prediction. Within the vitality classification model, the most influential bands were identified as 945.38 nm, 977.32 nm, 942.73 nm, 953.35 nm, and 937.42 nm, while the crucial bands for moisture prediction were 785.71 nm, 811.58 nm, 752.27 nm, 1004.07 nm, and 814.17 nm. Furthermore, employing the PCA technique, the first principal components for each respective task, PC1-classify and PC1-moisture, were extracted.

**Figure 10:**
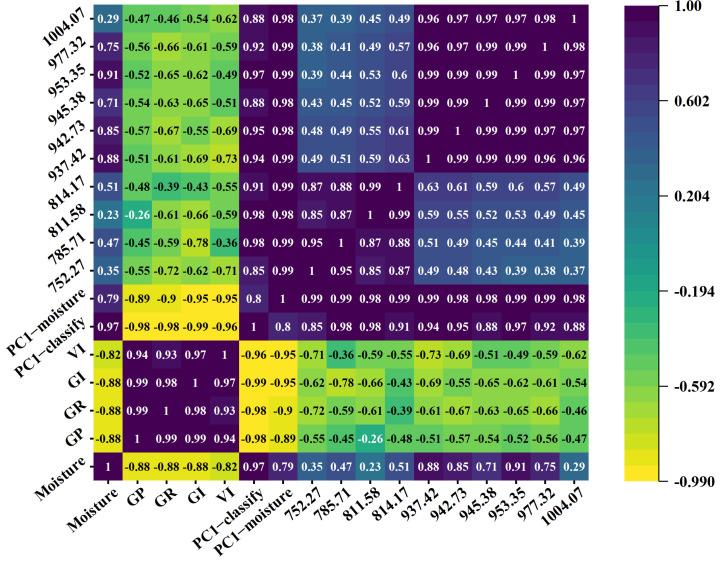
Characteristic band, activity index, and moisture content heat map.

Subsequently, five distinct aging levels of sunflower seeds (NAA, 1AA, 2AA, 3AA, and 4AA) were examined, calculating their average spectral reflectance and moisture content. Correlation analysis was then conducted to explore the relationships between sunflower seed vitality indices (including GP, GR, GI, and VI), average spectral reflectance, average moisture content, the top five feature band weights for vitality classification and moisture prediction, and their corresponding first principal components. Figure 10 illustrates a correlation heat map, which clearly displays highly positive correlations between seed vitality and growth indices, with correlation coefficients exceeding 0.9. Additionally, notable associations were found between bands such as 752.27 nm, 937.42 nm, 942.73 nm, 945.38 nm, 953.35 nm, and 977.32 nm with sunflower seed vitality indices, exhibiting a correlation coefficient of approximately 0.5. This aligns with prior discussions on feature bands relevant to sunflower seed vitality classification. Furthermore, significant correlations were observed between different feature bands and sunflower seed moisture content, with the highest correlation coefficient reaching 0.91, consistent with earlier results from moisture content regression analysis. Of particular significance is the remarkably strong correlation (0.88) between sunflower seed moisture content and vitality indices, highlighting the fundamental connection between moisture levels and seed vitality.

## 4. Conclusions

Seed vitality plays a pivotal role throughout the life cycle of crops, and its close association with seed moisture content is well recognized. In this study, leveraging the potential of hyperspectral imaging technology, we successfully evaluated the vitality of sunflower seeds and made accurate predictions regarding their internal moisture content. Following the completion of standard germination tests on sunflower seeds, relevant features, spectral bands, moisture content, and vitality indicators were meticulously collected for subsequent correlation analysis.

By comparing the performance of various classification models, the SG-SAE-LightGBM model stands out as the optimal choice for categorizing sunflower seed vitality, exhibiting an impressive accuracy of 97.67% on the training set and an outstanding 98.65% on the test set. Regarding the analysis of moisture content regression, the SNV-XGBoost-LightGBM model showcases remarkable results, with a correlation coefficient ($R_{c}^{2}$) of 0.9605 and a root mean square error of calibration (RMSEC) of 0.9776 on the training set, along with a correlation coefficient ($R_{p}^{2}$) of 0.9715 and a root mean square error of prediction (RMSEP) of 0.8349 on the prediction set.

The correlation analysis reveals a significant association between sunflower seed moisture content and vitality index, as demonstrated by the considerably high correlation coefficient of 0.88. These findings emphasize the influential role of moisture content on seed vitality.

In conclusion, this study successfully employs hyperspectral imaging technology to not only precisely discriminate vitality indicators of sunflower seeds but also effectively predict their internal moisture content. Moreover, it sheds light on the strong correlation between moisture content and vitality index, thereby providing a novel research perspective and methodology for future seed quality assessments.

## Figures and Tables

**Table 1:** Results of seed vigor index test.

Aging Class	Sample Count	Seed Vigor	Seed Inviability	GP	GR	GI	VI
NAA	49	43	6	81.63%	87.76%	14.510	120.433
1AA	47	33	14	59.57%	70.21%	9.641	53.026
2AA	50	25	25	40.00%	50.00%	5.996	28.781
3AA	50	18	32	20.00%	36.00%	3.769	14.699
4AA	50	10	40	8.00%	20.00%	1.926	6.934

**Table 2:** Statistical summary of moisture content in sunflower seeds after the removal of outliers.

Sample Type	Sample Size	Moisture Content (%)			
		Minimum	Maximum	Mean	Standard Deviation
Moisture content determination	236 grains	4.4799	24.6695	14.8082	4.9405

**Table 3:** Accuracy of sunflower seed vigor classification model.

Pretreatment	Feature Dimension Reduction	RF		LightGBM	
		Train	Test	Train	Test
RAW	All band	98.84%	91.89%	94.77%	94.59%
	PCA	95.93%	85.14%	97.67%	95.95%
	XGBoost	99.42%	90.54%	94.77%	95.95%
	SAE	97.09%	86.49%	97.09%	95.95%
SG	All band	99.42%	93.24%	94.19%	95.95%
	PCA	95.35%	86.49%	94.19%	95.95%
	XGBoost	98.84%	97.30%	95.35%	93.24%
	SAE	98.26%	87.84%	97.67%	98.65%
SNV	All band	97.09%	63.51%	89.53%	63.51%
	PCA	88.37%	64.86%	80.81%	60.81%
	XGBoost	95.93%	66.22%	86.63%	68.92%
	SAE	93.02%	47.30%	88.37%	60.81%
MSC	All band	96.51%	62.16%	88.37%	63.51%
	PCA	87.21%	67.57%	83.14%	60.81%
	XGBoost	94.19%	60.81%	87.79%	67.57%
	SAE	93.60%	51.35%	85.47%	62.16%

**Table 4:** Modeling results of sunflower seed moisture content regression model.

Pretreatment	Reduction Dimension	RF				LightGBM			
		$R_{c}^{2}$	RMSEC	$R_{p}^{2}$	RMSEP	$R_{c}^{2}$	RMSEC	$R_{p}^{2}$	RMSEP
RAW	All band	0.9183	1.4063	0.8644	1.8226	0.9415	1.1894	0.9416	1.1952
	PCA	0.9325	1.2778	0.8878	1.6572	0.8889	1.6394	0.9047	1.5271
	XGBoost	0.9344	1.2601	0.9012	1.5554	0.9320	1.2830	0.9438	1.1735
	SAE	0.8936	1.6044	0.8354	2.0075	0.9403	1.2016	0.9515	1.0889
SG	All band	0.9151	1.4335	0.8699	1.7847	0.9345	1.2591	0.9383	1.2285
	PCA	0.9275	1.3247	0.8824	1.6969	0.8889	1.6394	0.9031	1.5401
	XGBoost	0.9283	1.3171	0.8888	1.6498	0.9358	1.2458	0.9401	1.2111
	SAE	0.9164	1.4222	0.8684	1.7950	0.9210	1.3826	0.9191	1.4072
SNV	All band	0.8849	1.6692	0.9080	1.5004	0.9213	1.3803	0.9476	1.1319
	PCA	0.8650	1.8075	0.8790	1.7208	0.8458	1.9320	0.8515	1.9067
	XGBoost	0.8903	1.6293	0.9071	1.5082	0.9605	0.9776	0.9715	0.8349
	SAE	0.8569	1.8607	0.8688	1.7921	0.9156	1.4291	0.9321	1.2891
MSC	All band	0.8903	1.6297	0.9043	1.5306	0.9221	1.3727	0.9348	1.2633
	PCA	0.8684	1.7847	0.8874	1.6605	0.8452	1.9355	0.8548	1.8857
	XGBoost	0.8855	1.6645	0.9109	1.4769	0.9547	1.0468	0.9521	1.0058
	SAE	0.8803	1.7023	0.8911	1.6328	0.9166	1.4208	0.9392	1.2192

## Data Availability

The original contributions presented in the study are included in the article, further inquiries can be directed to the corresponding author.
